# Coronavirus disease 2019-related myocarditis genes contribute to ECMO prognosis

**DOI:** 10.1186/s12872-024-04032-5

**Published:** 2024-07-19

**Authors:** An Yan, Ruiying Zhang, Chao Feng, Jinping Feng

**Affiliations:** 1 Tianjin Chest Hospital, Taierzhuang North Road 261, Jinnan District, Tianjin, China; 2 Tianjin Key Laboratory of Cardiovascular Emergency and Critical Care, Taierzhuang North Road 261, Jinnan District, Tianjin, China

**Keywords:** Myocarditis, Single-cell sequencing, Pathway enrichment analysis, ECMO, Integrin, TNF family

## Abstract

**Background:**

Acute myocardial injury, cytokine storms, hypoxemia and pathogen-mediated damage were the major causes responsible for mortality induced by coronavirus disease 2019 (COVID-19)-related myocarditis. These need ECMO treatment. We investigated differentially expressed genes (DEGs) in patients with COVID-19-related myocarditis and ECMO prognosis.

**Methods:**

GSE150392 and GSE93101 were analyzed to identify DEGs. A Venn diagram was used to obtain the same transcripts between myocarditis-related and ECMO-related DEGs. Enrichment pathway analysis was performed and hub genes were identified. Pivotal miRNAs, transcription factors, and chemicals with the screened gene interactions were identified. The GSE167028 dataset and single-cell sequencing data were used to validate the screened genes.

**Results:**

Using a Venn diagram, 229 overlapping DEGs were identified between myocarditis-related and ECMO-related DEGs, which were mainly involved in T cell activation, contractile actin filament bundle, actomyosin, cyclic nucleotide phosphodiesterase activity, and cytokine-cytokine receptor interaction. 15 hub genes and 15 neighboring DEGs were screened, which were mainly involved in the positive regulation of T cell activation, integrin complex, integrin binding, the PI3K-Akt signaling pathway, and the TNF signaling pathway. Data in GSE167028 and single-cell sequencing data were used to validate the screened genes, and this demonstrated that the screened genes CCL2, APOE, ITGB8, LAMC2, COL6A3 and TNC were mainly expressed in fibroblast cells; IL6, ITGA1, PTK2, ITGB5, IL15, LAMA4, CAV1, SNCA, BDNF, ACTA2, CD70, MYL9, DPP4, ENO2 and VEGFC were expressed in cardiomyocytes; IL6, PTK2, ITGB5, IL15, APOE, JUN, SNCA, CD83, DPP4 and ENO2 were expressed in macrophages; and IL6, ITGA1, PTK2, ITGB5, IL15, VCAM1, LAMA4, CAV1, ACTA2, MYL9, CD83, DPP4, ENO2, VEGFC and IL32 were expressed in vascular endothelial cells.

**Conclusion:**

The screened hub genes, IL6, ITGA1, PTK2, ITGB3, ITGB5, CCL2, IL15, VCAM1, GZMB, APOE, ITGB8, LAMA4, LAMC2, COL6A3 and TNFRSF9, were validated using GEO dataset and single-cell sequencing data, which may be therapeutic targets patients with myocarditis to prevent MI progression and adverse cardiovascular events.

**Supplementary Information:**

The online version contains supplementary material available at 10.1186/s12872-024-04032-5.

## Introduction

Coronavirus disease 2019 (COVID-19) has resulted in an anthropogenic pneumonia epidemic worldwide [1]. COVID-19 related cardiovascular complications are independent risk factors for mortality and require more attention [2, 3]. Acute myocardial injury [3], cytokine storms [4, 5], hypoxemia [6], and pathogen-mediated damage [7, 8] are the major causes of COVID-19 related mortality.

The morbidity and mortality of patients with COVID-19 were higher than those of patients with SARS in 2003, developing more severe complications within a week, such as acute respiratory distress syndrome (ARDS), septic shock, and multiple organ failure (MOF) [9]. According to the treatment experience of severe COVID-19 infection from the front line, to prevent patients develop COVID-19 related severe complications and related mechanical complications, the early introduction and early offline of ExtraCorporeal Organ Support (ECOS), such as extracorporeal membrane oxygenation (ECMO), can help increase the survival rate and improve prognosis. [10, 11]. In addition, ECMO installation and utilization increase inflammation and even induce cytokine storms by regulating C5a and C3a [12]. However, the exact mechanism of COVID-19 infection-related myocardial injury remains unclear, and the prognostic biomarkers of COVID-19 infected myocarditis patients who need ECMO remain unclear.

In this study, COVID-19 induced myocarditis related differential expressed genes (DEGs) were obtained (from GSE150392) and ECMO prognosis-related DEGs were obtained (from GSE93101). Venn diagrams were used to obtain the same transcripts, and further enrichment pathway and protein-protein interaction (PPI) network analyses were performed to explore the hub genes. To validate the hub genes, GSE167028 for children with myocarditis and COVID-19 infection and single-cell sequencing were used.

## Methods

### Microarray data and data processing

Using the keyword “COVID-19,” GSE150392 from the Gene Expression Omnibus (GEO) database was downloaded [13]. RNA-sequencing of SARS-CoV-2 infected hiPSC-CMs and mock hiPSC-CMs was performed using an Illumina NextSeq 500 (Homo sapiens).

Using the keyword “ECMO,” GSE93101 was downloaded from the GEO database. The RNA-sequencing data included myocarditis patients who survived and died after ECMO installation and were based on the Illumina HumanHT-12 WG-DASL V4.0 R2 expression bead chip.

The data were analyzed using “DESeq2” (version 1.28.1) package in R. An adjusted P value < 0.05 and a Log2|FC (Fold Change) | >1 were considered as the cut-off criteria. Venn diagrams were used to obtain the same DEGs between COVID-19 related DEGs and ECMO prognosis-related DEGs, which may be potential prognostic biomarkers for myocarditis patients infected with COVID-19 and receiving ECMO.

### Enrichment pathways analysis

GO and KEGG pathway analysis were used to explore the functions of DEGs and Gene Set Enrichment Analysis (GSEA) was applied utilizing Xiantao Database (www.xiantao.love) [14–16]. An adjusted P value < 0.05 was considered as the cut-off criterion.

### PPI and the hub genes

To investigate the hub genes of the DEGs, the STRING database (https://string-db.org) was used with a combined score > 0.4 [17] and the nodes were analyzed using Cytoscape v.3.7.1 [18]. Hub genes were identified using the Cytoscape plug-in, MCODE. The Cytoscape plug-in Cytohubba was used to obtain the genes in the top15 MCC, top15 DMNC and top15 Degree. GO/KEGG pathway analysis were used to explore the functions of hub genes.

### The hub genes and their interactions

Hub genes and their interactions were analyzed using NetworkAnalyst 3.0 [19]. Specifically, transcription factor (TF) and gene target data were derived from the ENCODE ChIP-seq data, and the data with a peak intensity signal < 500 and predicted regulatory potential score < 1 were used. miRNA interactions with the screened hub genes were identified using miRTarBase v8.0. Hub DEMRG-chemical interactions are shown based on data from the Comparative Toxicogenomics Database (CTD)

### Validation

To validate the hub gene effects on cardiac function, GSE167028 was downloaded and analyzed using the Xiaotao website tool, which contains bulk-RNA-seq data on PBMC from nine children with COVID-19 infection and seven controls without COVID-19 infection [20].

To further validate which cells expressed the hub genes and whether hub gene expression and cytokine storms exacerbates COVID-19 related myocarditis after ECMO, single-cell sequencing data of COVID-19 heart autopsy samples were obtained from the Single Cell Portal (Study: SCP1216, https://singlecell.broadinstitute.org/) [21].

## Results

### Identification of DEGs and further analysis in COVID-19 related myocarditis

**Fig. 1:**
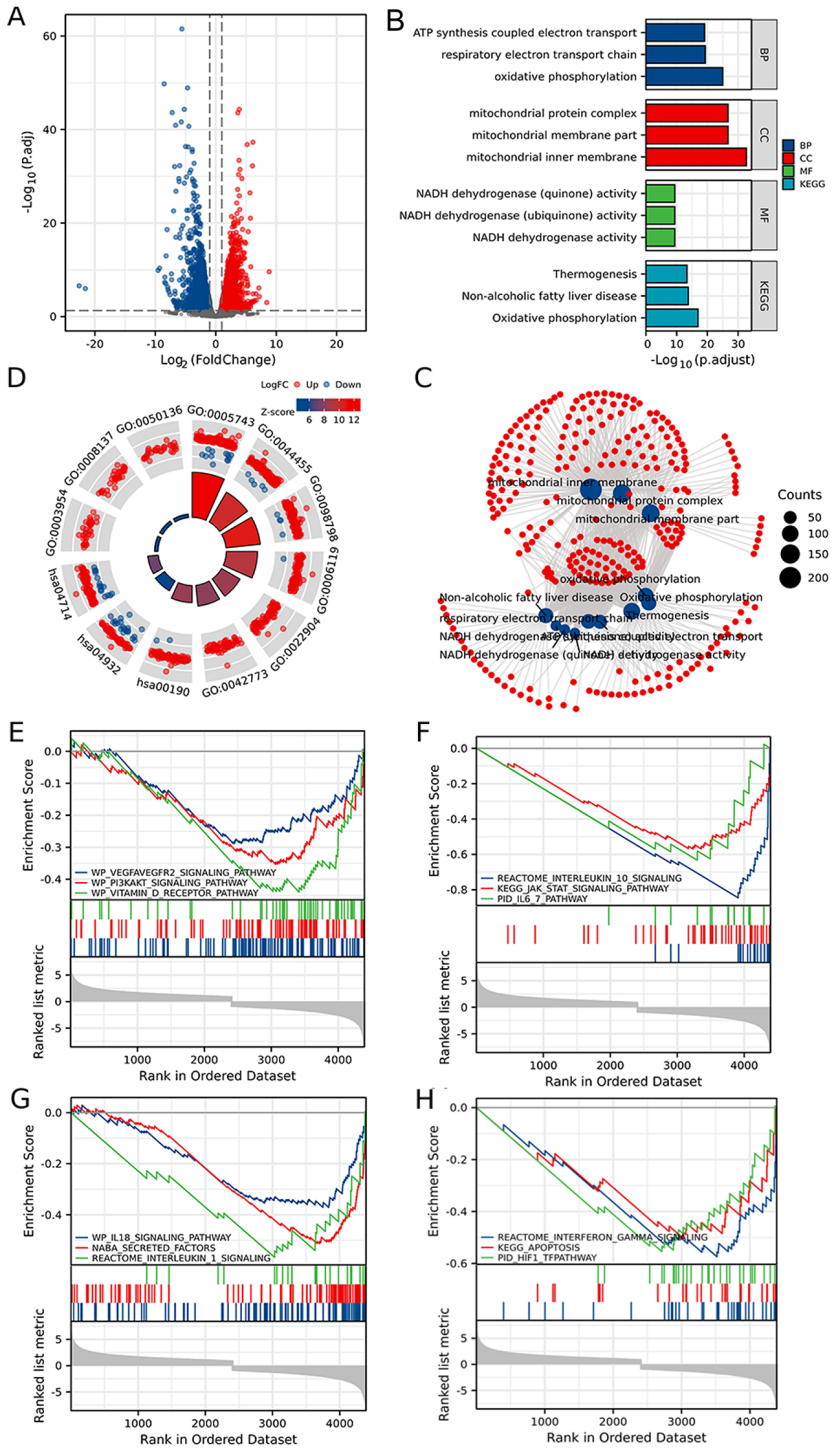
DEGs from myocarditis patients with COVID-19 infection. (**A**) The volcano plot of DEGs from myocarditis patients with COVID-19 infection. (**B**-**C**) The bar plot (**B**) and network (**C**) of the GO/KEGG pathways enriched by DEGs from myocarditis patients with COVID-19 infection. (**D**) The GO enrichment pathways of DEGs with log_2_FC parameter. (**E**-**H**) The GSEA of DEGs from myocarditis patients with COVID-19 infection

A total of 4,972 DEGs were obtained from GSE150392, which were mainly involved in the mitochondrial protein complex, NADH dehydrogenase (ubiquinone) activity, and oxidative phosphorylation (Fig. 1A-D; Table S1). GSEA demonstrated that the DEGs in COVID-19 related myocarditis were mainly involved in the REACTOME_INTERLEUKIN_1_SIGNALING, WP_IL18_SIGNALING_PATHWAY, WP_VEGFAVEGFR2_SIGNALING_PATHWAY, WP_VITAMIN_D_RECEPTOR_PATHWAY, and REACTOME_INTERLEUKIN_10_SIGNALING (Fig. 1E-H; Table S2).

### Identification of DEGs and further analysis in myocarditis after ECMO utilization

**Fig. 2:**
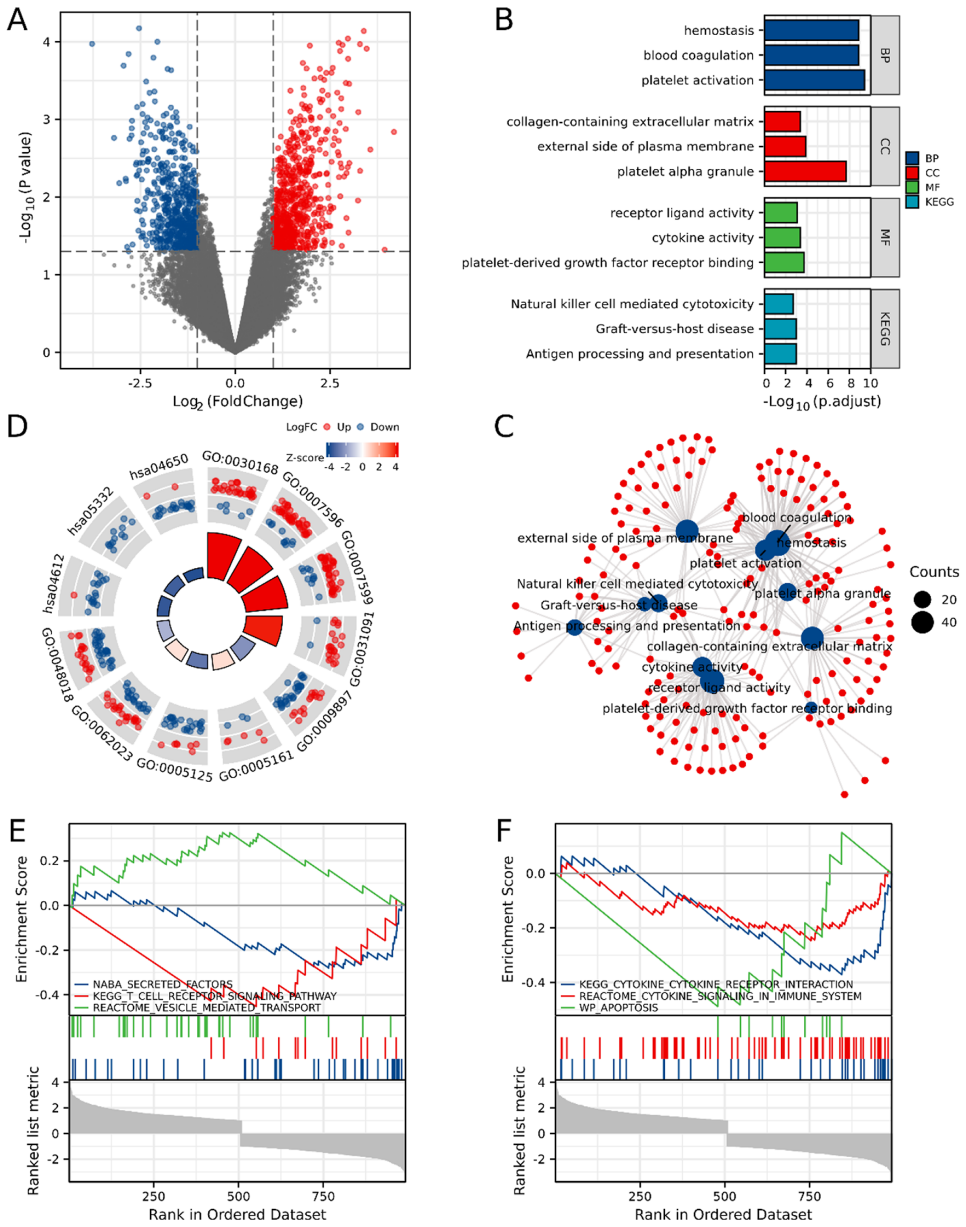
DEGs between survival and died myocarditis patients with ECMO installing. (**A**) The volcano plot of DEGs between survival and died myocarditis patients with ECMO installing. (**B**-**C**) The bar plot (**B**) and network (**C**) of the GO/KEGG pathways enriched by DEGs between survival and died myocarditis patients with ECMO installing. (**D**) The GO enrichment pathways of DEGs with log_2_FC parameter. (**E**-**F**) The GSEA of DEGs between survival and died myocarditis patients with ECMO installing

A total of 1,147 DEGs were obtained from GSE93101, which were mainly involved in blood coagulation, hemostasis, platelet-derived growth factor receptor binding, cytokine activity, and cytokine-cytokine receptor interactions (Fig. 2A-D; Table S3). GSEA results demonstrated that the DEGs between surviving and deceased myocarditis patients treated with ECMO were mainly involved in NABA_SECRETED_FACTORS, REACTOME_VESICLE_MEDIATED_TRANSPORT, and REACTOME_CYTOKINE_SIGNALING_IN_IMMUNE_SYSTEM (Fig. 2E, F; Table S4).

### The same transcripts between COVID-19 related DEGs and ECMO related DEGs

**Fig. 3:**
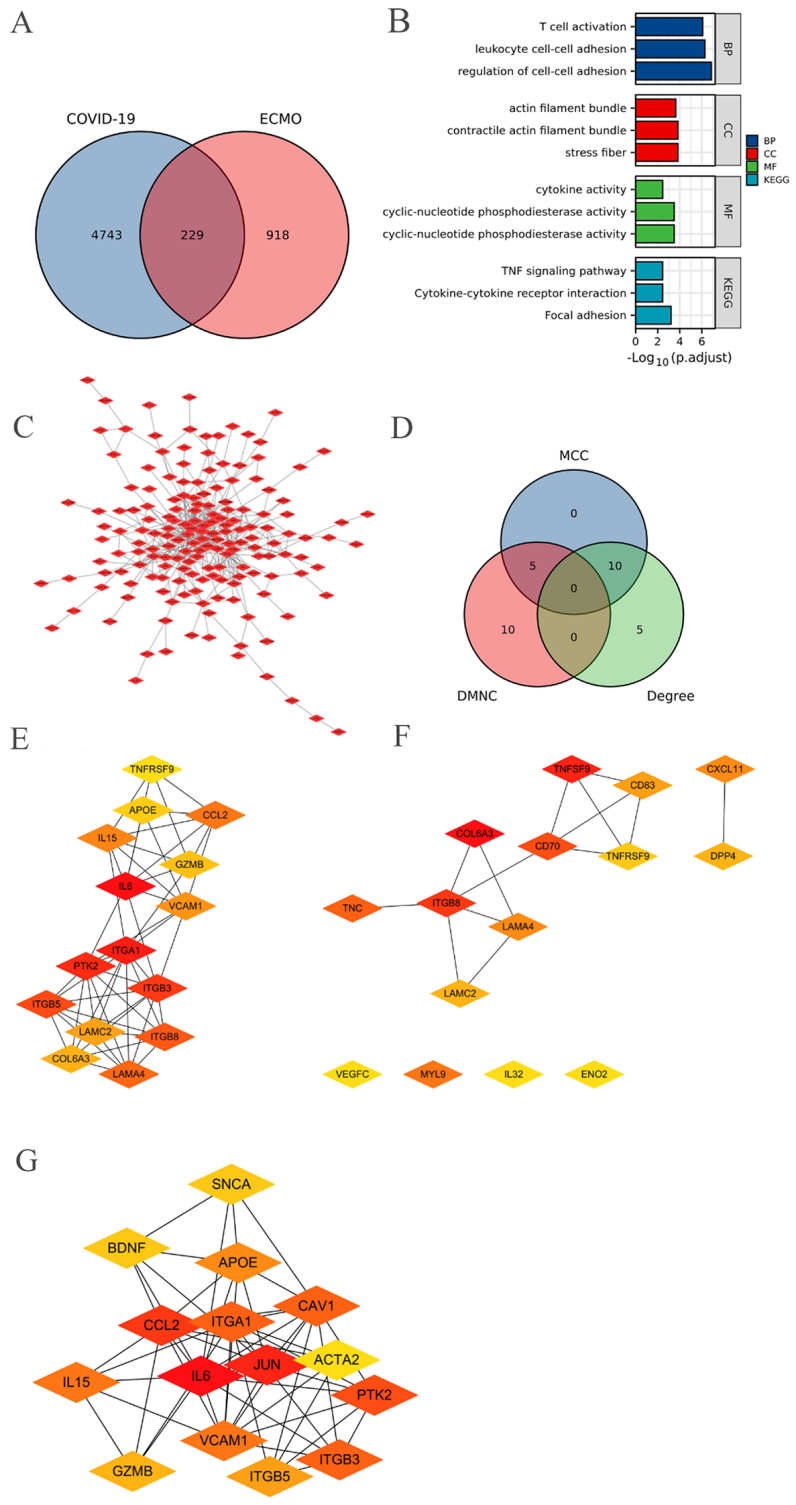
The same transcripts between COVID-19 related DEGs and ECMO prognostic related DEGs. (**A**) Venn diagram was used to obtain 229 the same transcripts. (**B**) The GO/KEGG pathways enriched by 229 DEGs. (**C**) The PPI network of 229 DEGs. (**D**) Venn diagram of the hub genes from Cytoscape plug-in MCC, DMNC and degree. (**E**-**G**) The network of the hub genes from Cytoscape plug-in MCC (**E**), DMNC (**F**) and degree (**G**)

Using a Venn diagram, 229 overlapping DEGs were obtained between COVID-19-related and ECMO prognosis-related DEGs. The GO/KEGG pathway analysis results demonstrated that these DEGs were mainly involved in T cell activation, contractile actin filament bundles, actomyosin, cyclic nucleotide phosphodiesterase activity, and cytokine-cytokine receptor interactions (Fig. 3A, B; Table 1).

**Table 1:** The GO/KEGG pathways enriched by 229 overlapping DEGs

ONTOLOGY	ID	Description	GeneRatio	BgRatio	pvalue	*p*.adjust	qvalue
BP	GO:0022407	regulation of cell-cell adhesion	24/213	403/18,670	4.04e-11	1.38e-07	1.12e-07
BP	GO:0007159	leukocyte cell-cell adhesion	21/213	337/18,670	3.15e-10	5.37e-07	4.36e-07
BP	GO:0042110	T cell activation	24/213	464/18,670	7.14e-10	8.11e-07	6.59e-07
BP	GO:1,903,037	regulation of leukocyte cell-cell adhesion	19/213	304/18,670	2.18e-09	1.86e-06	1.51e-06
BP	GO:0050863	regulation of T cell activation	19/213	314/18,670	3.72e-09	2.54e-06	2.06e-06
CC	GO:0001725	stress fiber	8/223	67/19,717	8.68e-07	1.43e-04	1.29e-04
CC	GO:0097517	contractile actin filament bundle	8/223	67/19,717	8.68e-07	1.43e-04	1.29e-04
CC	GO:0032432	actin filament bundle	8/223	75/19,717	2.08e-06	2.28e-04	2.06e-04
CC	GO:0042641	actomyosin	8/223	79/19,717	3.09e-06	2.30e-04	2.09e-04
CC	GO:0005925	focal adhesion	17/223	405/19,717	3.91e-06	2.30e-04	2.09e-04
MF	GO:0004114	3’,5’-cyclic-nucleotide phosphodiesterase activity	6/222	28/17,697	1.09e-06	3.16e-04	2.83e-04
MF	GO:0004112	cyclic-nucleotide phosphodiesterase activity	6/222	29/17,697	1.36e-06	3.16e-04	2.83e-04
MF	GO:0005125	cytokine activity	12/222	220/17,697	2.26e-05	0.004	0.003
MF	GO:0048018	receptor ligand activity	17/222	482/17,697	1.29e-04	0.015	0.013
MF	GO:0005126	cytokine receptor binding	12/222	286/17,697	2.75e-04	0.026	0.023
KEGG	hsa04510	Focal adhesion	14/127	201/8076	2.91e-06	6.13e-04	5.66e-04
KEGG	hsa04060	Cytokine-cytokine receptor interaction	15/127	295/8076	5.60e-05	0.004	0.003
KEGG	hsa04668	TNF signaling pathway	9/127	112/8076	6.22e-05	0.004	0.003
KEGG	hsa04512	ECM-receptor interaction	8/127	88/8076	6.72e-05	0.004	0.003
KEGG	hsa05410	Hypertrophic cardiomyopathy	7/127	90/8076	5.10e-04	0.022	0.020

DEGs, Different Expressed Genes; GO, Gene ONTOLOGY; BP, Biological Process; CC, cellular component; MF, Molecular Function; KEGG, Kyoto Encyclopedia of Genes and Genomes

A PPI network and Cytoscape plug-in (MCC, DNMC, Degree and MCODE methods) were used to screen the hub genes of the 229 overlapping DEGs. 30 DEGs were screened, including 15 intersected DEGs named hub genes (IL6, ITGA1, PTK2, ITGB3, ITGB5, CCL2, IL15, VCAM1, GZMB, APOE, ITGB8, LAMA4, LAMC2, COL6A3 and TNFRSF9) and other 15 neighbor genes (JUN, CAV1, SNCA, BDNF, ACTA2, TNFSF9, CD70, TNC, MYL9, CXCL11, CD83, DPP4, ENO2, VEGFC and IL32) (Fig. 3C-G; Fig. S1; Table 2).

**Table 2:** The information of the screened 30 genes

Gene symbol	Full name
**The hub genes**	
IL6	interleukin 6
ITGA1	integrin subunit alpha 1
PTK2	protein tyrosine kinase 2
ITGB3	integrin subunit beta 3
ITGB5	integrin subunit beta 5
CCL2	C-C motif chemokine ligand 2
IL15	interleukin 15
VCAM1	vascular cell adhesion molecule 1
GZMB	granzyme B
APOE	apolipoprotein E
ITGB8	integrin subunit beta 8
LAMA4	laminin subunit alpha 4
LAMC2	laminin subunit gamma 2
COL6A3	collagen type VI alpha 3 chain
TNFRSF9	TNF receptor superfamily member 9
**The neighbor genes**	
JUN	“Jun proto-oncogene, AP-1 transcription factor subunit”
CAV1	caveolin 1
SNCA	synuclein alpha
BDNF	brain derived neurotrophic factor
ACTA2	“actin alpha 2, smooth muscle”
TNFSF9	TNF superfamily member 9
CD70	CD70 molecule
TNC	tenascin C
MYL9	myosin light chain 9
CXCL11	C-X-C motif chemokine ligand 11
CD83	CD83 molecule
DPP4	dipeptidyl peptidase 4
ENO2	enolase 2
VEGFC	vascular endothelial growth factor C
IL32	interleukin 32

### The correlation among the 15 hub genes and 15 neighbor genes

**Fig. 4:**
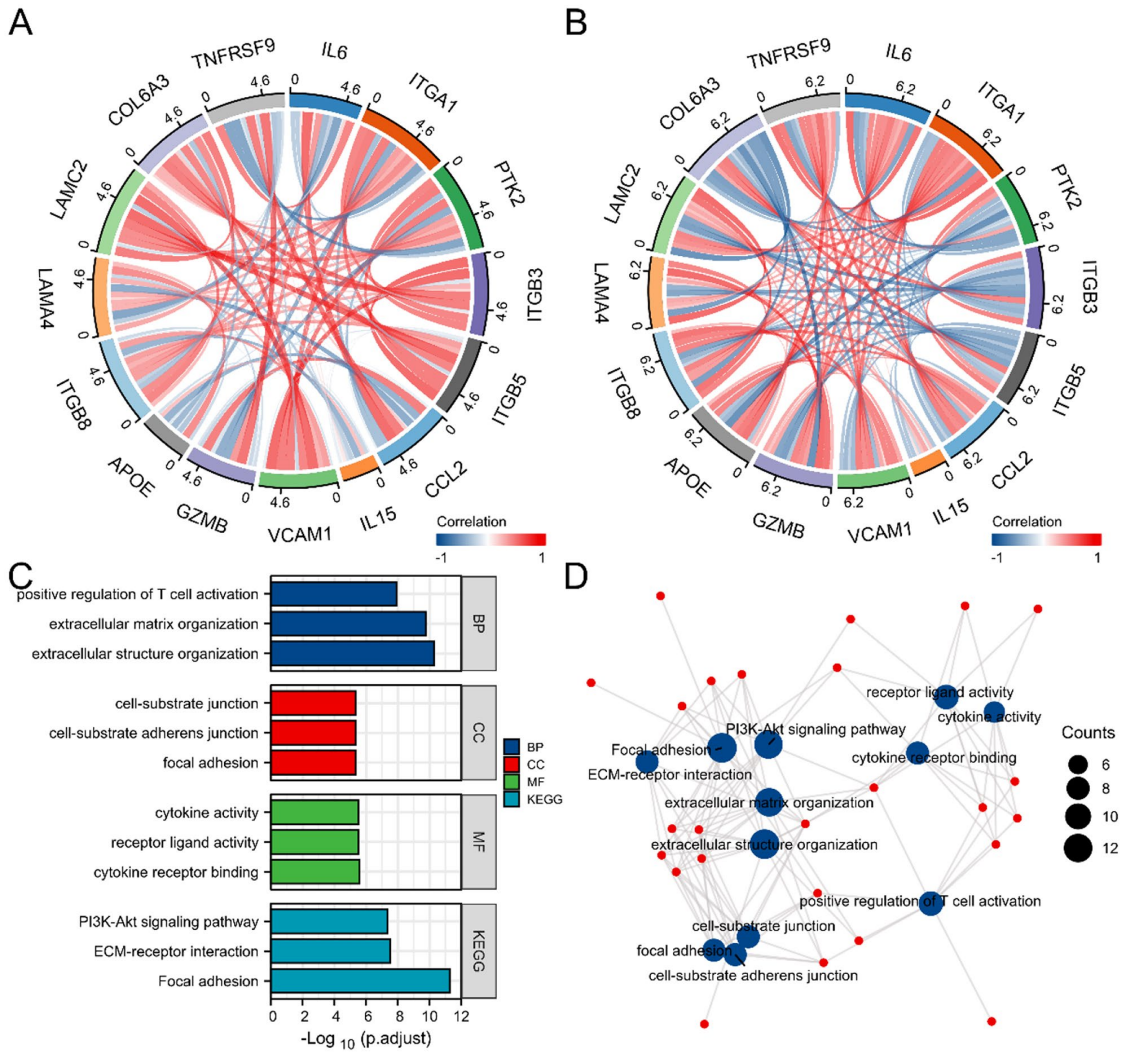
The hub genes enticement analysis. (**A**-**B**) The correlation among the 15 hub genes was constructed in COVID-19 related DEGs (**A**) and ECMO prognostic related DEGs (**B**). (**C**-**D**) The bar plot (**C**) and network (**D**) of the GO/KEGG pathways enriched by the 15 hub genes and 15 neighbor genes

Correlations among the 15 hub genes and 15 neighboring genes were analyzed in the GSE150392 and GSE93101 datasets (Fig. 4A, B; Fig. S2). The GO/KEGG enrichment pathway analysis results demonstrated that the screened genes were mainly involved in the positive regulation of T cell activation, integrin complex formation, integrin binding, the PI3K-Akt signaling pathway, and the TNF signaling pathway (Fig. 4C, D; Table S5).

### The screened DEGs and their interactions

**Fig. 5:**
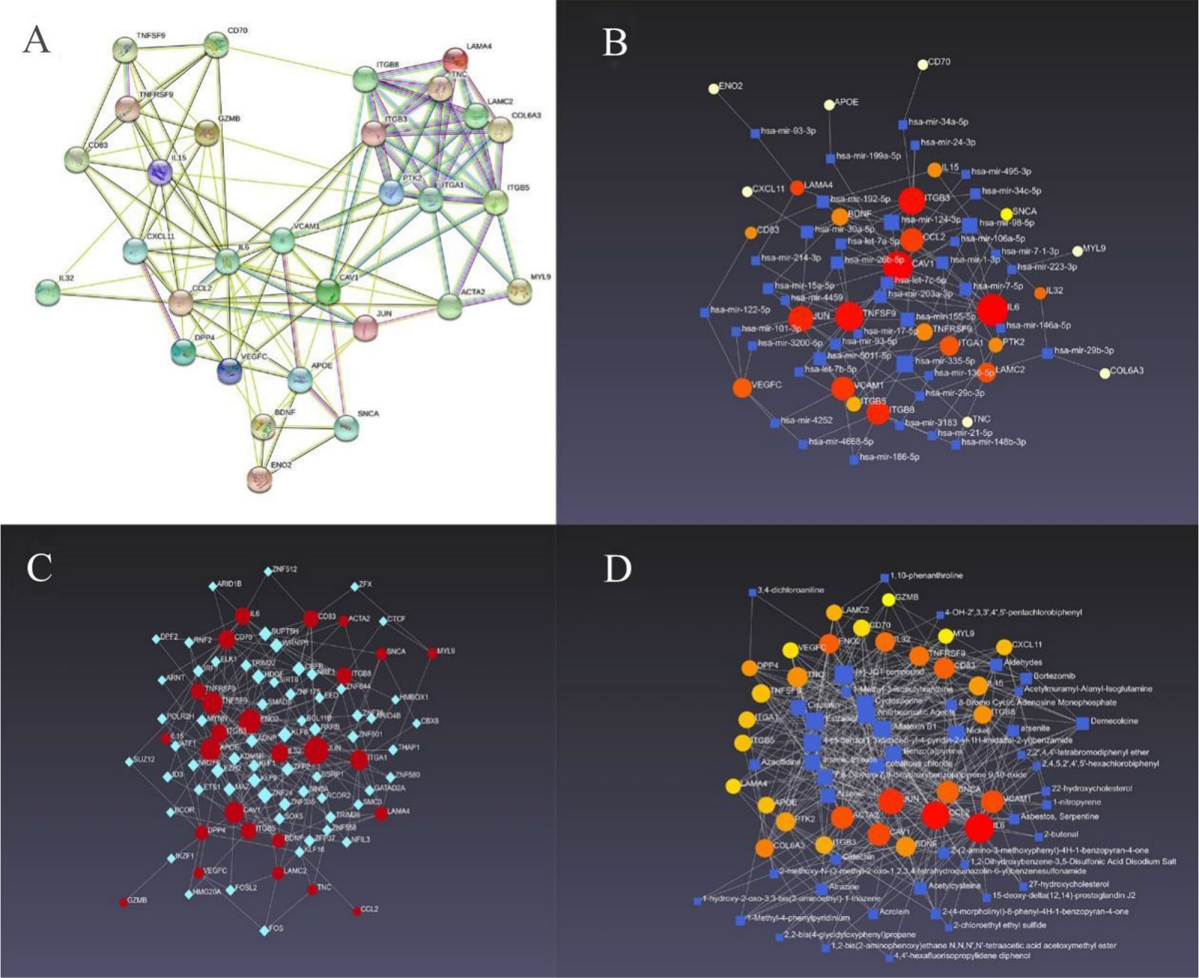
The screened DEGs and their interactions. (**A**) The PPI network of 30 screened DEGs. (**B**-**D**) TFs-the screened DEGs (**B**), miRNA-the screened DEGs (**C**) and 30 DEGs-chemicals networks (**D**) were constructed, which can further provide the insight for treatment to patients with COVID-19 infection and ECMO installing

The screened DEGs and their interactions are shown (Fig. 5). The screened DEGs, miRNA-screened DEGs, and 30 DEG-chemical networks were constructed, which further provided insight into the treatment of myocarditis patients with COVID-19 infection and ECMO installation.

### Validation

To validate the hub gene effects on cardiac function, GSE167028 was downloaded and analyzed. Compared with the control data, LAMA4 and VEGFC were highly expressed in children with myocarditis and COVID-19, whereas APOE and TNFRSF9 were expressed at low levels (Fig. S3).

**Fig. 6:**
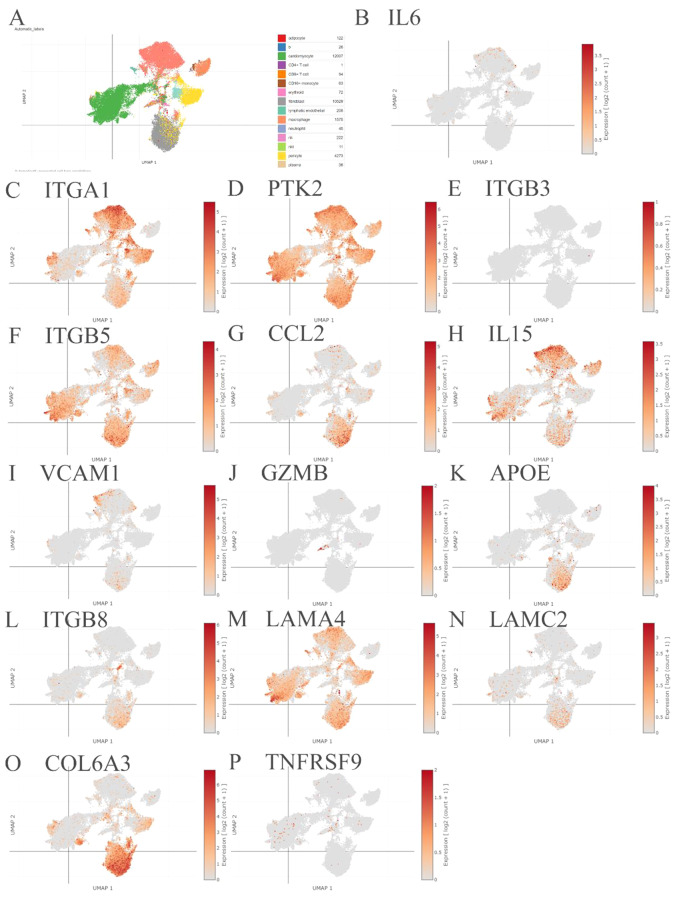
The single-cell sequencing validation of the hub genes. (**A**) The single-cell sequencing atlas of cell types from died patients with COVID-19 induced myocarditis. (**B**-**P**) The single-cell sequencing validation of the hub genes, including IL6 (**B**), ITGA1 (**C**), PTK2 (**D**), ITGB3 (**E**), ITGB5 (**F**), CCL2 (**G**), IL15 (**H**), VCAM1 (**I**), GZMB (**J**), APOE (**K**), ITGB8 (**L**), LAMA4 (**M**), LAMC2 (**N**), COL6A3 (**O**) and TNFRSF9 (**P**)

**Fig. 7:**
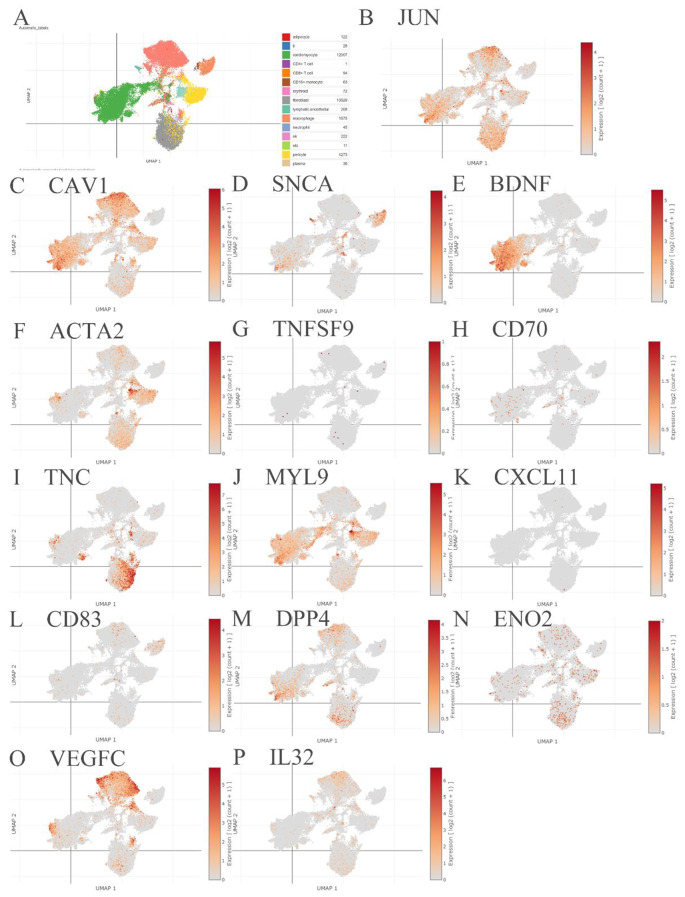
The single-cell sequencing validation of the neighbor genes. (**A**) The single-cell sequencing atlas of cell types from died patients with COVID-19 induced myocarditis. (**B**-**P**) The single-cell sequencing validation of the neighbor genes, including JUN (**B**), CAV1 (**C**), SNCA (**D**), BDNF (**E**), ACTA2 (**F**), TNFSF9 (**G**), CD70 (**H**), TNC (**I**), MYL9 (**J**), CXCL11 (**K**), CD83 (**L**), DPP4 (**M**), ENO2 (**N**), VEGFC (**O**) and IL32 (**P**)

Using the Single Cell Portal database, single-cell sequencing data were investigated to validate the hub gene effects, which may be potential targets for treatment (Figs. 6 and 7). Briefly, the screened genes, CCL2, APOE, ITGB8, LAMC2, COL6A3, and TNC were mainly expressed in fibroblasts. The screened genes IL6, ITGA1, PTK2, ITGB5, IL15, LAMA4, CAV1, SNCA, BDNF, ACTA2, CD70, MYL9, DPP4, ENO2 and VEGFC were expressed in cardiomyocytes; the screened genes, including IL6, PTK2, ITGB5, IL15, APOE, JUN, SNCA, CD83, DPP4 and ENO2 were expressed in macrophages; and the screened genes, including IL6, ITGA1, PTK2, ITGB5, IL15, VCAM1, LAMA4, CAV1, ACTA2, MYL9, CD83, DPP4, ENO2, VEGFC and IL32, were expressed in vascular endothelial cells (Table 3).

**Table 3:** The cells and expressed genes using single-cell sequencing

Fibroblasts	Cardiomyocytes	Macrophages	Vascular endothelial cells
*CCL2* *APOE* *ITGB8* *LAMC2* *COL6A3* *TNC*	*IL6* *ITGA1* *PTK2* *ITGB5* *IL15* *LAMA4* *CAV1* *SNCA* *BDNF* *ACTA2* *CD70* *MYL9* *DPP4* *ENO2* *VEGFC*	*IL6* *PTK2* *ITGB5* *IL15* *APOE* *JUN* *SNCA* *CD83* *DPP4* *ENO2*	*IL6* *ITGA1* *PTK2* *ITGB5* *IL15* *VCAM1* *LAMA4* *CAV1* *ACTA2* *MYL9* *CD83* *DPP4* *ENO2* *VEGFC* *IL32*

## Discussion

Angiotensin-converting enzyme 2 (ACE2) plays a critical role in SARS-CoV and SARS-CoV-2 infections, and is mainly expressed in the heart samples [22]. COVID-19 has spread widely among the population, leading to potentially fatal diseases [23, 24]. The long-term impact of COVID-19 induced myocarditis and myocardial injury, including most mild cases, requires further surveillance by survivors [25, 26]. An intra-aortic balloon pump (IABP) plus ECMO should be introduced earlier if the ejection fraction decreases quickly or heart failure appeared [27, 28]. In the present study, COVID-19 induced myocarditis related DEGs were obtained and ECMO-related prognostic DEGs were identified. A Venn diagram was used to obtain the 229 same transcripts and GSE167028 for children with myocarditis with COVID-19 infection, and single-cell sequencing of heart biopsy of COVID-19 induced myocarditis patients was utilized for the validation of the screened genes.

The enrichment pathway analysis of the 229 overlapping DEGs mainly involved T cell activation, contractile actin filament bundles, actomyosin, cyclic-nucleotide phosphodiesterase activity, and cytokine-cytokine receptor interactions, which is consistent with previous reports [29–31].

30 screened DEGs were identified, including 15 intersecting hub genes (IL6, ITGA1, PTK2, ITGB3, ITGB5, CCL2, IL15, VCAM1, GZMB, APOE, ITGB8, LAMA4, LAMC2, COL6A3, and TNFRSF9) and 15 neighboring genes (JUN, CAV1, SNCA, BDNF, ACTA2, TNFSF9, CD70, TNC, MYL9, CXCL11, CD83, DPP4, ENO2, VEGFC, and IL32). COVID-19 can induce cytokine storms, such as IL6, IL32, CD83, CCL2, CXCL11, the TNF family (TNFSF9 and the receptor TNFRSF9), and integrin-related genes, which exacerbates COVID-19 related myocarditis and decreases the prognosis of patients requiring ECMO treatment [32–34]. Overall, myocardial macrophage and lymphocyte densities were positively associated with symptom duration and myocarditis was more prevalent with IL-6 blockade than with non-biological immunosuppression [35, 36]. In this study, IL-6 expression decreased in heart biopsy samples from patients with COVID-19 infection, which may be a prognostic target for patients with COVID-19 myocarditis who require ECMO treatment. Integrin-related genes, including ITGA1, ITGB3, ITGB5, and ITGB8, have been less investigated in patients with COVID-19 myocarditis and may be potential prognostic biomarkers. Tenascin-C, a nonstructural extracellular matrix glycoprotein expressed during heart injury and remodeling, aggravates autoimmune myocarditis via dendritic cell activation and Th17 cell differentiation [37, 38]. Two DPP-4 inhibitors suppress fibrosis and inflammation in experimental autoimmune myocarditis in mice [39, 40]. ICAM-1 and VCAM-1 are persistently expressed in the myocardial tissue of children with lymphocytic myocarditis [41]. IL-15 promotes atherogenesis and IL-15 played a critical role in the treatment of cardiovascular diabetology [42].

In this study, based on single-cell sequencing, the screened genes, CCL2, APOE, ITGB8, LAMC2, COL6A3, and TNC, were mainly expressed in fibroblasts from heart biopsies with COVID-19 infection. The screened genes IL6, ITGA1, PTK2, ITGB5, IL15, LAMA4, CAV1, SNCA, BDNF, ACTA2, CD70, MYL9, DPP4, ENO2, and VEGFC were expressed in cardiomyocytes, whereas the screened genes IL6, PTK2, ITGB5, IL15, APOE, JUN, SNCA, CD83, DPP4, and ENO2 were expressed in macrophages. In addition, IL6, ITGA1, PTK2, ITGB5, IL15, VCAM1, LAMA4, CAV1, ACTA2, MYL9, CD83, DPP4, ENO2, VEGFC and IL32, were expressed in the vascular endothelial cells, which can be referred to as the mechanical investigation of the cell interplay in COVID-19 myocarditis. In addition, treatment with the screened hub genes may help decrease acute myocarditis inflammation following mRNA COVID-19 vaccination, thus increasing patients’ prognosis [43–46].

The current study had several limitations. The study included available data on hiPSC-CMs and ECMO samples, which could not completely reflect the effect of COVID-19 infection with ECMO installed on the heart in vivo. Only bioinformatic analysis was conducted in this study, and further clinical reports or in vitro studies are required to support our conclusions. The absence of corresponding molecular biology experiments has limited our findings, and each biomarker requires validation and qualification before clinical application. After validation and qualification, ELISA kits for these hub genes may be used to predict the prognosis of myocarditis patients who require ECMO and guide the best ECMO weaning time. Additionally, ELISA kits for these hub genes may also be used to predict the prognosis of patients following mRNA COVID-19 vaccination.

## Conclusion

Based on our current study, our research provided bioinformatics analysis about the prognostic targets for patients with COVID-19 myocarditis who need ECMO treatment. The screened hub genes, including IL6, ITGA1, PTK2, ITGB3, ITGB5, CCL2, IL15, VCAM1, GZMB, APOE, ITGB8, LAMA4, LAMC2, COL6A3, and TNFRSF9, were validated using the GEO dataset and single-cell sequencing data, and may be therapeutic targets in patients with COVID-19 myocarditis to prevent myocarditis progression and adverse cardiovascular events.

### Electronic supplementary material

Below is the link to the electronic supplementary material.

Supplementary Material 1

## Data Availability

Previously reported GEO data were used to support this study and are available at GSE150392, GSE93101 and GSE167028. These datasets are cited at relevant places within the text as references [13, 20, 21].
