# Supplementary material for: Coronavirus disease 2019-related myocarditis genes contribute to ECMO prognosis
Source: BMC Cardiovasc Disord. 2024 Jul 19;24:375. doi: 10.1186/s12872-024-04032-5 (PMC11264513; doi:10.1186/s12872-024-04032-5)
Supplement: Supplementary file 1 — Supplementary Material 1 [file 12872_2024_4032_MOESM1_ESM.docx]

**Supplementary Figure Legend**


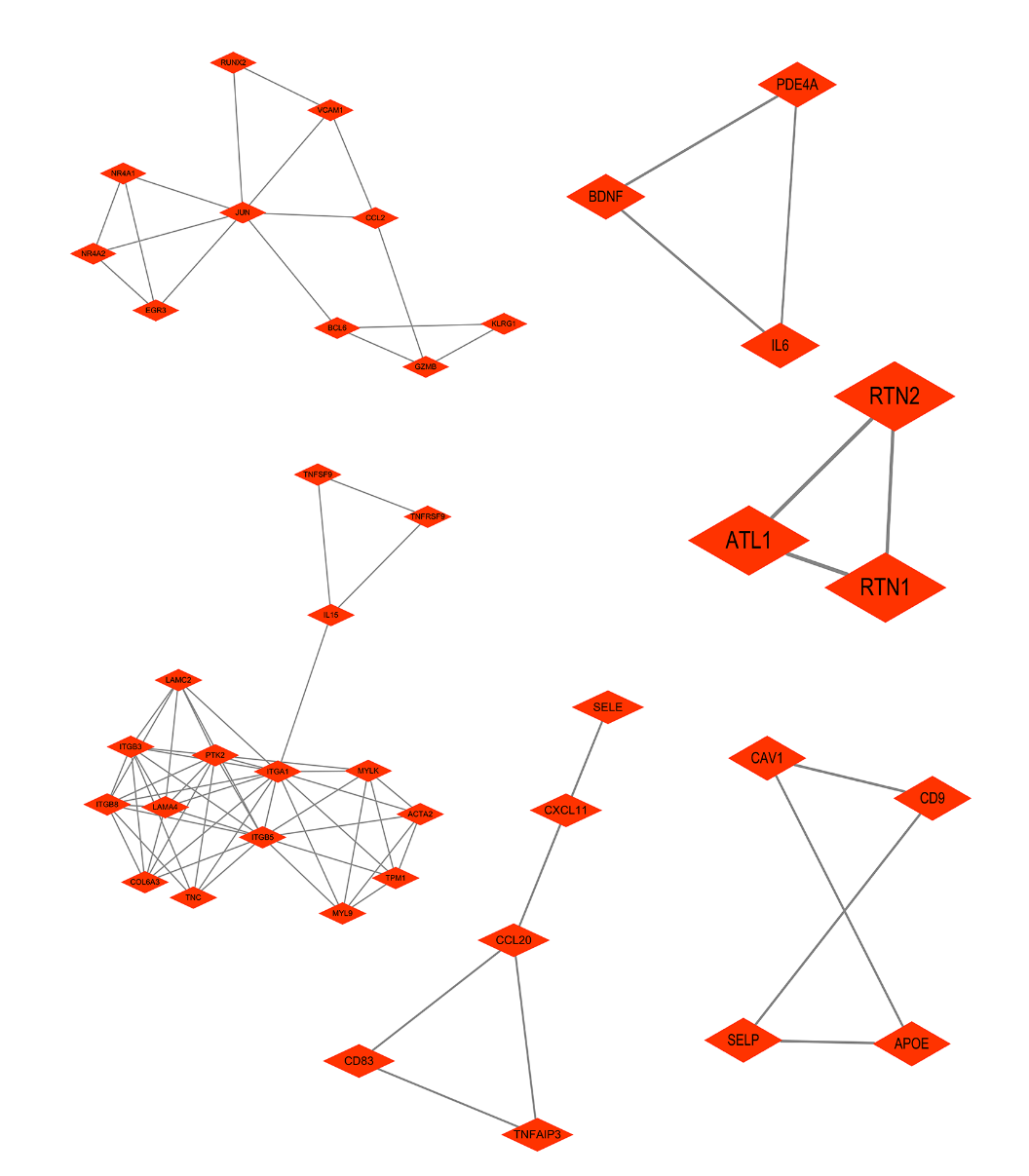


Supplementary Figure 1. The 6 modules of 229 DEGs using Cytpscape plug-in MCODE.


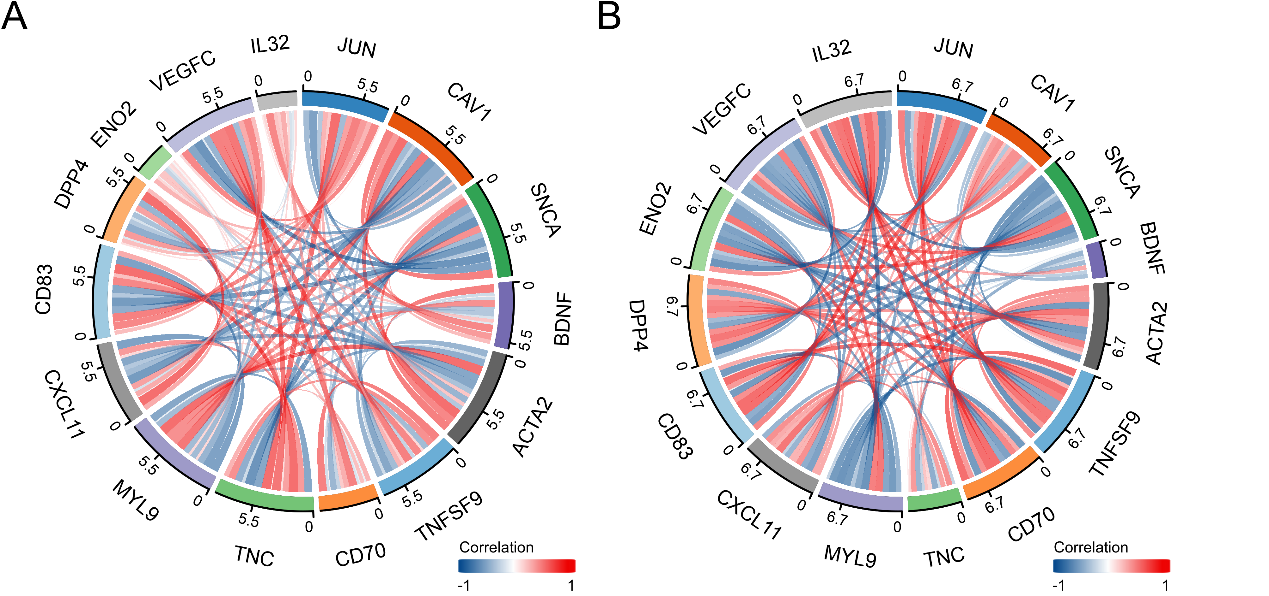


Supplementary Figure 2. The correlation among the 15 neighbor genes was constructed in COVID-19 related DEGs (A) and ECMO prognostic related DEGs (B).


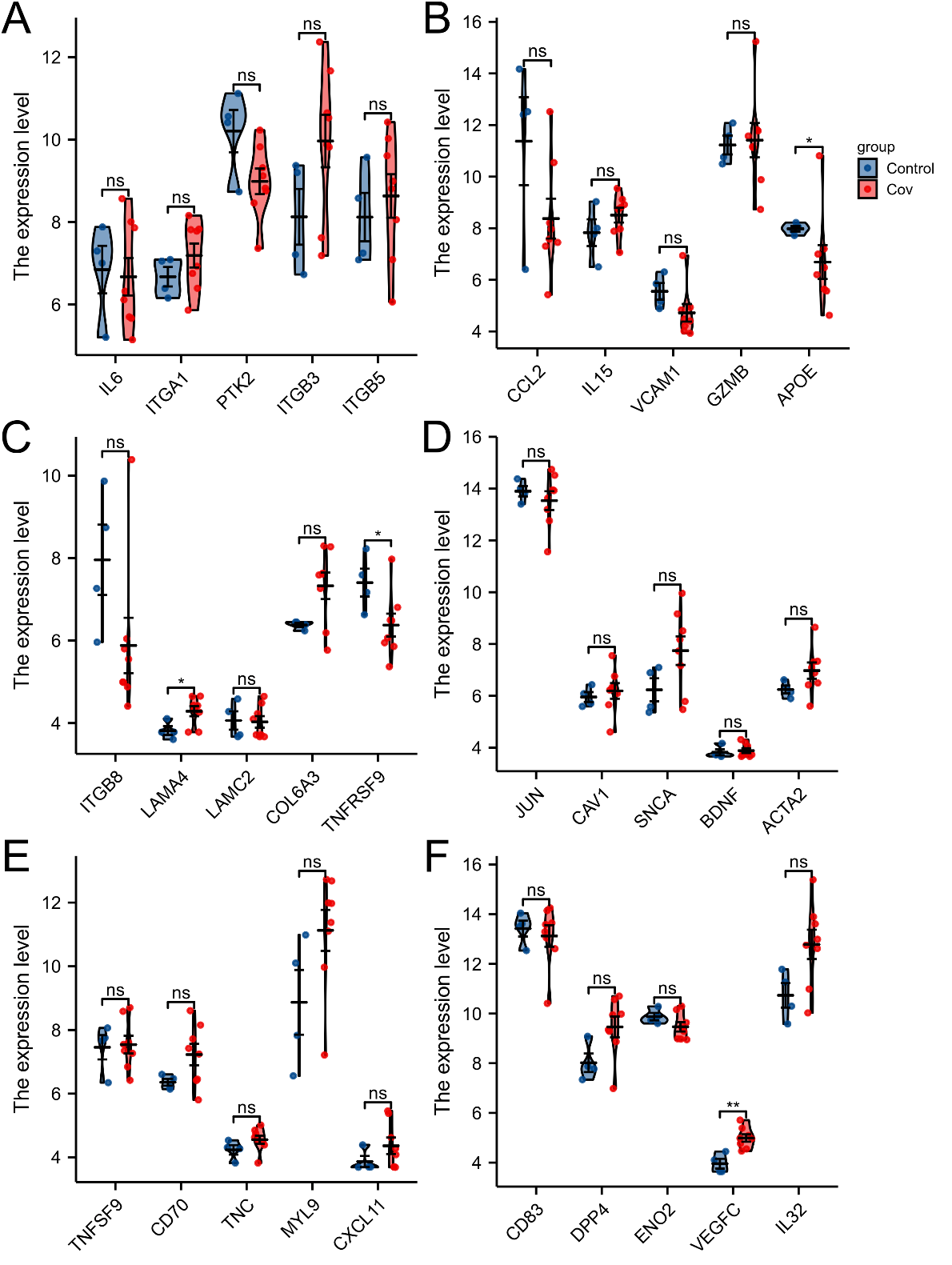


Supplementary Figure 3. The validation of the screened DEGs. (A-F) GSE167028 were downloaded and analyzed, demonstrating LAMA4 and VEGFC were highly expressed in myocarditis children with COVID-19 infection, while APOE and TNFRSF9 were lowly expressed.

Supplementary Table 1. The GO/KEGG pathways enriched by DEGs from myocarditis patients with COVID-19 infection.

| ONTOLOGY | ID | Description | GeneRatio | BgRatio | pvalue | p.adjust | qvalue |
| --- | --- | --- | --- | --- | --- | --- | --- |
| BP | GO:0006119 | oxidative phosphorylation | 90/3627 | 145/18670 | 1.62e-29 | 1.03e-25 | 7.75e-26 |
| BP | GO:0022904 | respiratory electron transport chain | 72/3627 | 117/18670 | 1.47e-23 | 4.67e-20 | 3.51e-20 |
| BP | GO:0042773 | ATP synthesis coupled electron transport | 64/3627 | 98/18670 | 3.78e-23 | 8.02e-20 | 6.02e-20 |
| BP | GO:0045333 | cellular respiration | 98/3627 | 193/18670 | 1.09e-22 | 1.64e-19 | 1.23e-19 |
| BP | GO:0042775 | mitochondrial ATP synthesis coupled electron transport | 63/3627 | 97/18670 | 1.29e-22 | 1.64e-19 | 1.23e-19 |
| CC | GO:0005743 | mitochondrial inner membrane | 210/3814 | 473/19717 | 2.60e-36 | 1.96e-33 | 1.66e-33 |
| CC | GO:0044455 | mitochondrial membrane part | 121/3814 | 229/19717 | 5.89e-30 | 1.94e-27 | 1.65e-27 |
| CC | GO:0098798 | mitochondrial protein complex | 132/3814 | 262/19717 | 7.72e-30 | 1.94e-27 | 1.65e-27 |
| CC | GO:0098800 | inner mitochondrial membrane protein complex | 85/3814 | 135/19717 | 1.01e-28 | 1.91e-26 | 1.62e-26 |
| CC | GO:1990204 | oxidoreductase complex | 66/3814 | 112/19717 | 2.37e-20 | 3.59e-18 | 3.04e-18 |
| MF | GO:0003954 | NADH dehydrogenase activity | 32/3641 | 46/17697 | 1.04e-12 | 4.03e-10 | 3.59e-10 |
| MF | GO:0008137 | NADH dehydrogenase (ubiquinone) activity | 32/3641 | 46/17697 | 1.04e-12 | 4.03e-10 | 3.59e-10 |
| MF | GO:0050136 | NADH dehydrogenase (quinone) activity | 32/3641 | 46/17697 | 1.04e-12 | 4.03e-10 | 3.59e-10 |
| MF | GO:0016655 | oxidoreductase activity, acting on NAD(P)H, quinone or similar compound as acceptor | 37/3641 | 60/17697 | 4.89e-12 | 1.42e-09 | 1.26e-09 |
| MF | GO:0009055 | electron transfer activity | 50/3641 | 114/17697 | 1.58e-08 | 3.65e-06 | 3.25e-06 |
| KEGG | hsa00190 | Oxidative phosphorylation | 77/1733 | 133/8076 | 3.41e-20 | 1.10e-17 | 8.68e-18 |
| KEGG | hsa04932 | Non-alcoholic fatty liver disease | 78/1733 | 150/8076 | 1.03e-16 | 1.65e-14 | 1.31e-14 |
| KEGG | hsa04714 | Thermogenesis | 104/1733 | 231/8076 | 4.08e-16 | 4.38e-14 | 3.47e-14 |
| KEGG | hsa05020 | Prion disease | 104/1733 | 273/8076 | 1.41e-10 | 1.13e-08 | 8.95e-09 |
| KEGG | hsa05012 | Parkinson disease | 96/1733 | 249/8076 | 3.45e-10 | 2.22e-08 | 1.76e-08 |

DEGs, Different Expressed Genes; GO, Gene ONTOLOGY; BP, Biological Process; CC, cellular component; MF, Molecular Function; KEGG, Kyoto Encyclopedia of Genes and Genomes.

Supplementary Table 2. The GSEA of DEGs enriched by DEGs from myocarditis patients with COVID-19 infection.

| Description | setSize | | enrichmentScore | | NES | pvalue | p.adjust | qvalues | rank | leading_edge |
| --- | --- | --- | --- | --- | --- | --- | --- | --- | --- | --- |
| WP_VEGFAVEGFR2_SIGNALING_PATHWAY | | 117 | | -0.288549398 | -1.865600419 | 0.001675042 | 0.012649693 | 0.007253948 | 1942 | tags=72%, list=44%, signal=41% |
| WP_PI3KAKT_SIGNALING_PATHWAY | | 85 | | -0.353756612 | -2.128111067 | 0.001692047 | 0.012649693 | 0.007253948 | 1328 | tags=54%, list=30%, signal=38% |
| WP_VITAMIN_D_RECEPTOR_PATHWAY | | 51 | | -0.440438305 | -2.361134585 | 0.001692047 | 0.012649693 | 0.007253948 | 978 | tags=69%, list=22%, signal=54% |
| REACTOME_INTERLEUKIN_10_SIGNALING | | 21 | | -0.845666259 | -3.451877187 | 0.001763668 | 0.012649693 | 0.007253948 | 483 | tags=86%, list=11%, signal=77% |
| KEGG_JAK_STAT_SIGNALING_PATHWAY | | 43 | | -0.569509692 | -2.888361861 | 0.001785714 | 0.012649693 | 0.007253948 | 1156 | tags=70%, list=26%, signal=52% |
| PID_IL6_7_PATHWAY | | 14 | | -0.632753127 | -2.230707677 | 0.001766784 | 0.012649693 | 0.007253948 | 1094 | tags=79%, list=25%, signal=59% |
| WP_IL18_SIGNALING_PATHWAY | | 99 | | -0.371131418 | -2.307356328 | 0.001718213 | 0.012649693 | 0.007253948 | 556 | tags=34%, list=13%, signal=31% |
| NABA_SECRETED_FACTORS | | 94 | | -0.512679313 | -3.143004855 | 0.001730104 | 0.012649693 | 0.007253948 | 695 | tags=50%, list=16%, signal=43% |
| REACTOME_INTERLEUKIN_1_SIGNALING | | 23 | | -0.566405715 | -2.402396234 | 0.001733102 | 0.012649693 | 0.007253948 | 1362 | tags=78%, list=31%, signal=54% |
| REACTOME_INTERFERON_GAMMA_SIGNALING | | 37 | | -0.574507709 | -2.818481904 | 0.001736111 | 0.012649693 | 0.007253948 | 875 | tags=59%, list=20%, signal=48% |
| KEGG_APOPTOSIS | | 27 | | -0.502149192 | -2.240668005 | 0.001754386 | 0.012649693 | 0.007253948 | 1373 | tags=70%, list=31%, signal=49% |
| PID_HIF1_TFPATHWAY | | 27 | | -0.554636281 | -2.474873581 | 0.001754386 | 0.012649693 | 0.007253948 | 1680 | tags=89%, list=38%, signal=55% |

Supplementary Table 3. The GO/KEGG pathways enriched by DEGs between survival and died myocarditis patients with ECMO treatment.

| ONTOLOGY | ID | Description | GeneRatio | BgRatio | pvalue | p.adjust | qvalue |
| --- | --- | --- | --- | --- | --- | --- | --- |
| BP | GO:0030168 | platelet activation | 34/914 | 153/18670 | 7.15e-14 | 3.84e-10 | 3.18e-10 |
| BP | GO:0007596 | blood coagulation | 51/914 | 336/18670 | 5.28e-13 | 1.42e-09 | 1.17e-09 |
| BP | GO:0007599 | hemostasis | 51/914 | 341/18670 | 9.44e-13 | 1.42e-09 | 1.18e-09 |
| BP | GO:0050817 | coagulation | 51/914 | 342/18670 | 1.06e-12 | 1.42e-09 | 1.18e-09 |
| BP | GO:0022407 | regulation of cell-cell adhesion | 55/914 | 403/18670 | 5.15e-12 | 5.53e-09 | 4.58e-09 |
| CC | GO:0031091 | platelet alpha granule | 23/946 | 91/19717 | 3.32e-11 | 1.96e-08 | 1.69e-08 |
| CC | GO:0009897 | external side of plasma membrane | 43/946 | 393/19717 | 4.25e-07 | 1.25e-04 | 1.08e-04 |
| CC | GO:0062023 | collagen-containing extracellular matrix | 42/946 | 406/19717 | 2.51e-06 | 4.16e-04 | 3.60e-04 |
| CC | GO:0031093 | platelet alpha granule lumen | 14/946 | 67/19717 | 2.82e-06 | 4.16e-04 | 3.60e-04 |
| CC | GO:0005925 | focal adhesion | 40/946 | 405/19717 | 1.31e-05 | 0.001 | 0.001 |
| MF | GO:0005161 | platelet-derived growth factor receptor binding | 8/903 | 15/17697 | 2.08e-07 | 1.92e-04 | 1.81e-04 |
| MF | GO:0005125 | cytokine activity | 30/903 | 220/17697 | 8.76e-07 | 4.05e-04 | 3.80e-04 |
| MF | GO:0048018 | receptor ligand activity | 49/903 | 482/17697 | 3.40e-06 | 8.58e-04 | 8.05e-04 |
| MF | GO:0005126 | cytokine receptor binding | 34/903 | 286/17697 | 4.02e-06 | 8.58e-04 | 8.05e-04 |
| MF | GO:0008009 | chemokine activity | 12/903 | 49/17697 | 4.64e-06 | 8.58e-04 | 8.05e-04 |
| KEGG | hsa04612 | Antigen processing and presentation | 17/512 | 78/8076 | 5.56e-06 | 0.001 | 9.22e-04 |
| KEGG | hsa05332 | Graft-versus-host disease | 12/512 | 42/8076 | 7.10e-06 | 0.001 | 9.22e-04 |
| KEGG | hsa04650 | Natural killer cell mediated cytotoxicity | 22/512 | 131/8076 | 2.20e-05 | 0.002 | 0.002 |
| KEGG | hsa01521 | EGFR tyrosine kinase inhibitor resistance | 16/512 | 79/8076 | 2.79e-05 | 0.002 | 0.002 |
| KEGG | hsa04060 | Cytokine-cytokine receptor interaction | 37/512 | 295/8076 | 4.58e-05 | 0.003 | 0.002 |

DEGs, Different Expressed Genes; GO, Gene ONTOLOGY; BP, Biological Process; CC, cellular component; MF, Molecular Function; KEGG, Kyoto Encyclopedia of Genes and Genomes.

Supplementary Table 4. The GSEA enriched by DEGs between survival and died myocarditis patients with ECMO treatment.

| Description | setSize | enrichmentScore | NES | pvalue | p.adjust | qvalues | rank | leading_edge |
| --- | --- | --- | --- | --- | --- | --- | --- | --- |
| NABA_SECRETED_FACTORS | 42 | -0.281503997 | -1.77949732 | 0.012019231 | 0.09527897 | 0.080234922 | 184 | tags=38%, list=19%, signal=32% |
| KEGG_T_CELL_RECEPTOR_SIGNALING_PATHWAY | 14 | -0.452406593 | -1.840832913 | 0.012875536 | 0.09527897 | 0.080234922 | 441 | tags=86%, list=44%, signal=48% |
| REACTOME_VESICLE_MEDIATED_TRANSPORT | 34 | 0.326540036 | 1.785725611 | 0.019332162 | 0.123325859 | 0.103853355 | 453 | tags=74%, list=46%, signal=41% |
| KEGG_CYTOKINE_CYTOKINE_RECEPTOR_INTERACTION | 38 | -0.371509569 | -2.267507297 | 0.002257336 | 0.032387955 | 0.027274068 | 148 | tags=42%, list=15%, signal=37% |
| REACTOME_CYTOKINE_SIGNALING_IN_IMMUNE_SYSTEM | 70 | -0.246856558 | -1.803874854 | 0.007832898 | 0.072454308 | 0.061014154 | 239 | tags=40%, list=24%, signal=33% |
| WP_APOPTOSIS | 11 | -0.488277268 | -1.797707123 | 0.012578616 | 0.09527897 | 0.080234922 | 514 | tags=100%, list=52%, signal=49% |

Supplementary Table 5. The GO/KEGG pathways enriched by the screened 15 hub and 15 neighbor DEGs.

| ONTOLOGY | ID | Description | GeneRatio | BgRatio | pvalue | p.adjust | qvalue |
| --- | --- | --- | --- | --- | --- | --- | --- |
| BP | GO:0043062 | extracellular structure organization | 13/30 | 422/18670 | 2.83e-14 | 5.22e-11 | 3.10e-11 |
| BP | GO:0030198 | extracellular matrix organization | 12/30 | 368/18670 | 1.81e-13 | 1.67e-10 | 9.93e-11 |
| BP | GO:0050870 | positive regulation of T cell activation | 9/30 | 202/18670 | 2.00e-11 | 1.23e-08 | 7.31e-09 |
| BP | GO:1903039 | positive regulation of leukocyte cell-cell adhesion | 9/30 | 218/18670 | 3.96e-11 | 1.82e-08 | 1.09e-08 |
| BP | GO:0022409 | positive regulation of cell-cell adhesion | 9/30 | 255/18670 | 1.60e-10 | 5.90e-08 | 3.51e-08 |
| CC | GO:0005925 | focal adhesion | 8/30 | 405/19717 | 1.17e-07 | 4.46e-06 | 2.59e-06 |
| CC | GO:0005924 | cell-substrate adherens junction | 8/30 | 408/19717 | 1.23e-07 | 4.46e-06 | 2.59e-06 |
| CC | GO:0030055 | cell-substrate junction | 8/30 | 412/19717 | 1.33e-07 | 4.46e-06 | 2.59e-06 |
| CC | GO:0008305 | integrin complex | 4/30 | 31/19717 | 1.33e-07 | 4.46e-06 | 2.59e-06 |
| CC | GO:0098636 | protein complex involved in cell adhesion | 4/30 | 34/19717 | 1.96e-07 | 5.24e-06 | 3.05e-06 |
| MF | GO:0005126 | cytokine receptor binding | 8/30 | 286/17697 | 1.81e-08 | 2.56e-06 | 1.53e-06 |
| MF | GO:0048018 | receptor ligand activity | 9/30 | 482/17697 | 6.58e-08 | 3.13e-06 | 1.87e-06 |
| MF | GO:0005125 | cytokine activity | 7/30 | 220/17697 | 6.66e-08 | 3.13e-06 | 1.87e-06 |
| MF | GO:0005178 | integrin binding | 4/30 | 132/17697 | 6.97e-05 | 0.002 | 0.001 |
| MF | GO:0005201 | extracellular matrix structural constituent | 4/30 | 163/17697 | 1.58e-04 | 0.004 | 0.003 |
| KEGG | hsa04510 | Focal adhesion | 13/29 | 201/8076 | 4.57e-14 | 5.39e-12 | 3.75e-12 |
| KEGG | hsa04512 | ECM-receptor interaction | 8/29 | 88/8076 | 5.12e-10 | 3.02e-08 | 2.10e-08 |
| KEGG | hsa04151 | PI3K-Akt signaling pathway | 12/29 | 354/8076 | 1.11e-09 | 4.35e-08 | 3.03e-08 |
| KEGG | hsa05165 | Human papillomavirus infection | 9/29 | 331/8076 | 1.42e-06 | 4.18e-05 | 2.91e-05 |
| KEGG | hsa04668 | TNF signaling pathway | 6/29 | 112/8076 | 2.28e-06 | 5.38e-05 | 3.74e-05 |

DEGs, Different Expressed Genes; GO, Gene ONTOLOGY; BP, Biological Process; CC, cellular component; MF, Molecular Function; KEGG, Kyoto Encyclopedia of Genes and Genomes.
